# Determinants and prognostic implications of instantaneous wave-free ratio in patients with mild to intermediate coronary stenosis: Comparison with those of fractional flow reserve

**DOI:** 10.1371/journal.pone.0237275

**Published:** 2020-08-06

**Authors:** Kyohei Onishi, Heitaro Watanabe, Kazuyoshi Kakehi, Tomoyuki Ikeda, Toru Takase, Kenji Yamaji, Masafumi Ueno, Kazuhiro Kobuke, Gaku Nakazawa, Shunichi Miyazaki, Yoshitaka Iwanaga

**Affiliations:** 1 Division of Cardiology, Department of Internal Medicine, Kindai University Faculty of Medicine, Osakasayama, Japan; 2 Saiseikai-Tondabayashi Hospital, Tondabayasi, Japan; Universita degli Studi Magna Graecia di Catanzaro, ITALY

## Abstract

The instantaneous wave-free ratio (iFR) is used for assessing the hemodynamic severity of a lesion, as an alternative to the fractional flow reserve (FFR). We evaluated the relationship between iFR and FFR in detail and the clinical significance of iFR in patients with mild to intermediate coronary artery stenosis. We recruited consecutive 323 patients (421 lesions) with lesions exhibiting 30% to 80% diameter stenosis on angiography in whom FFR and iFR were measured. In the total lesions, mean diameter stenosis was 48.6% ± 9.0%, and physiological significance, defined by FFR of 0.80 or less or by iFR of 0.92 or less, was observed in 32.5% or 33.5%, respectively. Mismatch between iFR and FFR was observed in 18.1% of the lesions. Clinical factors did not predict FFR value; however, gender, diabetes mellitus, aortic stenosis, anemia, high-sensitivity CRP value, and renal function predicted iFR value. In multivariate logistic analysis after adjustment for FFR value, gender (*p* < 0.001), diabetes mellitus (*p* = 0.005), aortic stenosis (*p* = 0.016), high-sensitivity CRP (*p* < 0.001), and renal function (*p* = 0.003) were all independent predictors of iFR value. In Kaplan-Meier analysis, the baseline iFR predicted the subsequent major cardiovascular events (MACE) (hazard ratio, 2.40; 95% CI, 1.16–4.93; *p* = 0.018) and the results of the iFR-guided strategy for predicting rates of MACE and myocardial infarction/revascularization were superior to those of the FFR-guided strategy. In conclusion, significant clinical factors predicted iFR value, which affected the prognostic capacity. The iFR-guided strategy may be superior in patients with mild to intermediate stenosis.

## Introduction

Fractional flow reserve (FFR) is defined as the ratio of distal coronary pressure divided by the proximal one (aortic pressure) in the stenosis at maximal hyperemia. This condition is induced by administration of a vasodilator agent in order to identify coronary stenosis that can induce reversible myocardial ischemia [1]. The FFR optimizes the risk stratification of patients with chest pain who are undergoing coronary angiography (CAG), and this use of the FFR has been supported by results of several trials and guideline recommendations [2]. The instantaneous wave-free ratio (iFR) is a recently introduced physiological index assessing the severity of stenosis without the administration of a vasodilator agent. It is defined as the ratio of resting distal coronary pressure to aortic pressure during the period of diastole in which microvascular resistance is minimized and constant (wave-free period) [3]. FFR and iFR have been demonstrated to show no significant differences in the prediction of myocardial ischemia from nitrogen-13–ammonia positron emission tomography [4]. A meta-analysis has shown excellent agreement of iFR with FFR without the undesired effects and cost of hyperemic agents [5]. In addition, it is also comparable with FFR in guiding revascularization according to two large randomized controlled trials [6, 7]. However, FFR-iFR mismatch has been recognized, and the reason for these discrepancies and those in clinical and angiographic characteristics of discordant lesions is not fully understood [8]. Because iFR is measured during resting status, whereas FFR is measured during hyperemic status, each index must represent a different aspect of pathophysiology in patients with coronary artery disease (CAD), especially in intermediate coronary lesions [9].

Watanabe et al. recently reported that not only the extent of local stenosis but also the amount of myocardial supply and the lesion location determined the physiological significance and may explain the visual–functional mismatch between CAG and FFR in mild to intermediate coronary stenosis [10]. Therefore, we evaluated the relationship of iFR with FFR in detail, and we determined the predictors for iFR and its prognostic potentials in 323 consecutive patients with CAD and mild to intermediate coronary artery stenosis. We further examined whether iFR-guided strategy for predicting cardiac events was clinically reasonable in this setting.

## Materials and methods

### Study protocol

Consecutive patients with stable CAD were enrolled between November 2013 and March 2017 at Kindai Hospital (Osakasayama, Japan). Patients eligible to participate had a lesion angiographically determined to be 30% to 80% diameter stenosis (DS) [11] and had undergone invasive physiological assessment before percutaneous coronary intervention (PCI) or coronary artery bypass grafting (CABG). Patients were exluded when they had acute coronary syndrome, left main coronary artery stenosis, and coronary artery bypass grafted lesions. The measurements and analyses in quantitative coronary angiography (QCA), and iFR and the follow-up of clinical outcomes were performed retrospectively. The study was conducted in accordance with the 1964 Declaration of Helsinki and its later amendments and was approved by the Ethics Committee of Kindai University Faculty of Medicine, Osakasayama, Japan. The ethics committee waived the requirement for the informed consent and all data were fully anonymized before accessing them.

Aortic stenosis was defined as peak aortic velocity of 2.6 m/s or faster or an aortic valve area of less than 2.0 cm^2^ according to echocardiography. Plasma B-type natriuretic peptide, hemoglobin, serum high-sensitive C-reactive protein (hs-CRP), creatinine, and hemoglobin A1c levels were measured before CAG. The estimated glomerular filtration rate (eGFR) was calculated according to the equation specific to the Japanese population: eGFR = 194 × (serum creatinine) − 1.094 × (age) − 0.287 (× 0.739 for female patients).

### CAG and QCA

CAG was performed with a standard technique involving the transradial or transfemoral approach [10]. Patients were administered intracoronary isosorbide dinitrate (1 to 5 mg) before initial angiography to achieve maximal vasodilation. CAG images were reviewed by independent physicians who were unaware of patients’ clinical characteristics and FFR value. QCA was performed to locate FFR measurement with the use of CAAS II (Pie Medical Imaging, Maastricht, The Netherlands) by an independent experienced physician who was unaware of the FFR result and other data [12]. Measurements included the minimum lumen diameter, reference vessel diameter, and lesion length at the target coronary segment before FFR was calculated. Percentage DS was calculated as the ratio of the minimum lumen diameter to the reference vessel diameter. Diffuse lesion was defined as stenotic lesions that were 20 mm in length or longer.

To evaluate the myocardial area supplied by the coronary artery distal to the stenosis, a modified version of the Bypass Angioplasty Revascularization Investigation (BARI) score was utilized, as in a previous study [10].

### FFR and iFR measurements

Intracoronary pressure was measured with a 0.014-in. pressure guide wire (PressureWire; St. Jude Medical, St. Paul, MN, USA). It advanced to distal of the assessed area of stenosis. The proximal coronary pressure was measured via the guiding catheter. FFR was calculated as the mean distal coronary pressure divided by the mean aortic pressure at maximal hyperemia. Maximal hyperemia was induced by continuous intravenous infusion of adenosine 5´-triphosphate, administered at 140 μg/kg/min via the forearm vein in accordance with previous studies [13]. Subsequently, the pressure guide wire was manually pulled back slowly from the most distal part of the artery to the proximal part during induced steady-state maximal hyperemia in all patients. When the FFR value was 0.8 or lower, the coronary stenosis was considered functionally significant.

Coronary pressure recordings were extracted from a data storage system (RadiView, St. Jude Medical) and processed offline by our own algorithm. The iFR was defined as the ratio of distal coronary pressure to aortic pressure during the wave-free period (approximately 75% of late diastole) at rest [14]. We identified the dicrotic notch to recognize the onset of the diastolic phase, and the wave-free period (excluding the first 25% of diastole and ending 5 msec before the end of diastole) was evaluated.

### Clinical follow-up

To monitor long-term clinical outcomes after FFR/iFR testing, patients completed a questionnaire, a telephone interview, or a chart review. Major adverse cardiovascular events (MACEs) were defined as the combination of all-cause death, myocardial infarction (MI), and the need for emergent revascularization. A secondary outcome was defined as the combination of MI and need for emergent revascularization. Clinical outcomes of patients who did not undergo subsequent revascularization because FFR exceeded 0.80 (the “FFR-defer” group) were compared with those of patients who did because FFR was 0.8 or less (the “FFR-perform” group) [15]. They were also compared on the basis of iFR: patients who did not undergo subsequent revascularization because iFR exceeded 0.92 (the “iFR-defer” group) and those who did because iFR was 0.92 or less (the “iFR-perform” group).

### Statistical analysis

As part of the univariable analysis for continuous variables, comparisons among groups were performed with Student’s *t* test, one-way analysis of variance, and the Mann-Whitney *U* test. Pearson’s χ^2^ and Fisher’s exact tests were used to assess differences in categorical variables. A linear regression analysis with the Pearson correlation coefficient was used to assess the linearity of the relationship between two variables. Multiple logistic regression and univariable analyses were used to explore the significant predictors of FFR and iFR. Cutoff levels of iFR for physiological significance (FFR ≤ 0.8) and the sensitivities and specificities of the cutoff levels were calculated in receiver operating characteristics (ROC) curve analysis. Event-free survival curves were analyzed according to the Kaplan-Meier method, and curves were compared in log-rank tests. Multivariable analysis of clinical outcomes was performed with Cox’s proportional hazards model, with the use of JMP V.14.3 (SAS Institute, Cary, NC, USA). Hazard ratios (HRs) and 95% confidence intervals (CIs) were calculated. A *p* value of less than 0.05 was considered significant. All results are expressed as means ± standard deviations.

## Results

### Baseline patient and lesion characteristics

CAG and FFR examinations were performed in 351 patients with 465 lesions. Of the lesions, 14 were excluded because they were left main coronary artery lesions, and 2 were excluded because they were CABG lesions. Another 16 lesions were excluded because they represented either less than 30% or more than 80% DS on angiography. In 12 lesions, iFR data was unavailable for the post hoc analysis. A total of 323 patients with 421 lesions were included in the analysis (S1 Fig).

The baseline characteristics of the patients and lesions are listed in Table 1. The mean age of the patients was 70.6 ± 9.0 years, and 76.5% were male. Furthermore, 48.2% of the lesions were located in the left anterior descending coronary artery (LAD), and the remaining were in the left circumflex artery (LCX) and right coronary artery. The mean FFR value was 0.84 ± 0.09, and physiological significance as defined by FFR of 0.8 or less was observed in 32.5% of the lesions. The minimum lumen diameter and reference vessel diameter in lesions of the right coronary artery were significantly larger than those in the LAD or LCX. In addition, lower FFR value was observed in LAD lesions as compared with that in LCX lesions or lesions of the right coronary artery (*p* < 0.001).

**Table 1:** Patient and lesion characteristics.

Characteristic	Findings
*Patients (n = 323)*	
Age, years	70.6 ± 9.0
No. male	247 (76.5%)
BMI, kg/m^2^	23.9 ± 3.4
No. currently smoking	151 (46.7%)
No. with diabetes mellitus	172 (53.4%)
Hemoglobin level, %	6.4 ± 0.9
No. with hypertension	279 (86.6%)
No. with hypercholesterolemia	285 (88.5%)
LDL-cholesterol, mg/dL	91.0 ± 31.4
No. with chronic kidney disease	148 (46.1%)
eGFR, mL/min/1.73 m^2^	59.1 ± 22.1
No. on hemodialysis	25 (7.8%)
No. with anemia	26 (8.1%)
Hb, mg/dL	13.4 ± 1.6
Log (hs-CRP, mg/dL)	−2.28 ± 1.40
No. with aortic stenosis	23 (7.3%)
Peak aortic velocity, m/s	1.5 ± 0.6
No. with old myocardial infarction	156 (48.4%)
No. with prior PCI	218 (67.7%)
No. with prior CABG	10 (3.1%)
*Lesions (n = 421)*	
Location	
LAD	203 (48.2%)
Non-LAD	218 (51.8%)
Diffuse lesion	103 (24.5%)
QCA findings	
Lesion length, mm	10.8 ± 6.0
Minimum lumen diameter, mm	1.40 ± 0.41
Reference vessel diameter, mm	2.72 ± 0.65
DS, %	48.6 ± 9.0
FFR ≤ 0.80	137 (32.5%)
iFR ≤ 0.92	141 (33.5%)

Values are means ± standard deviations or numbers (%).

BMI, body mass index; CABG, coronary artery bypass grafting; DS, diameter stenosis; eGFR, estimated glomerular filtration rate; FFR, fractional flow reserve; Hb, hemoglobin; hs-CRP, high-sensitive C-reactive protein; iFR, instantaneous wave-free ratio; LAD, left anterior descending coronary artery; LDL, low-density lipoprotein; PCI, percutaneous coronary intervention; QCA, quantitative coronary angiography.

ROC analysis showed that the area under the ROC curve for iFR as an indicator of physiological significance (FFR ≤ 0.80) was 0.876 (S2 Fig). The optimal cutoff value of iFR was 0.92 (sensitivity, 74%; specificity, 86%). In this cohort, the mean iFR value was 0.94 ± 0.06, and physiological significance as defined by iFR of 0.92 or less was observed in 33.5% of the lesions.

### Relationship of iFR or FFR to patient and lesion characteristics

Although iFR was correlated with FFR (*R* = 0.709, *p* < 0.001; S3 Fig), mismatch between iFR and FFR (FFR > 0.80 and iFR ≤ 0.92 or FFR ≤ 0.80 and iFR > 0.92) was observed in 18.1% of the lesions. When the cutoff value of iFR was set to 0.89 [6, 7], the physiological significance defined by it and the mismatch between iFR and FFR were observed in 18.5% and 19.7%, respectively. The baseline clinical characteristics except hemoglobin A1c level did not predict the FFR value; however, the clinical factors such as gender, diabetes mellitus, aortic stenosis, anemia, hs-CRP levels, and renal function, predicted the iFR value, as shown in Table 2.

**Table 2:** Univariable analysis of FFR and iFR and baseline characteristics.

Characteristics	Relationship to FFR (*p*)	Relationship to iFR (*p*)
*Patients*		
Age	0.071	0.358
Gender	0.809	0.005
BMI	0.461	0.238
Currently smoking	0.785	0.599
Diabetes mellitus	0.149	<0.001
Hemoglobin A1c level	0.016	0.019
Hypertension	0.124	0.080
Hypercholesterolemia	0.259	0.202
LDL-cholesterol	0.978	0.929
Chronic kidney disease	0.423	0.133
eGFR	0.182	0.009
Hemodialysis	0.645	0.062
Anemia	0.212	0.008
Hemoglobin	0.865	0.002
Log hs-CRP	0.070	<0.001
Aortic stenosis	0.177	<0.001
Peak aortic velocity	0.165	<0.001
*Lesions*		
Location*	< 0.001	< 0.001
Diffuse lesion	< 0.001	< 0.001
QCA findings		
Lesion length	< 0.001	0.046
Minimum lumen diameter	< 0.001	< 0.001
Reference vessel diameter	< 0.001	< 0.001
DS	< 0.001	< 0.001
BARI score	<0.001	<0.001

*Lesion location indicates the distribution ratio of LAD to non-LAD.

BARI, Bypass Angioplasty Revascularization Investigation; BMI, body mass index; DS, percent diameter stenosis; eGFR, estimated glomerular filtration rate; FFR, fractional flow reserve; hs-CRP, high-sensitive C-reactive protein; iFR, instantaneous wave-free ratio; LAD, left anterior descending coronary artery; LDL, low-density lipoprotein; QCA, quantitative coronary angiography.

The lesion characteristics, such as minimum lumen diameter, lesion location (LAD versus LCX or right coronary artery), diffuse lesion, proximal lesion, and BARI score FFR, was independently associated with FFR (fit of the model: *R*^*2*^ = 0.536) [10]; those lesion characteristics was similarly associated with iFR (fit of the model: *R*^*2*^ = 0.391). Hemoglobin A1c level was not associated with FFR independently with the multivariate analysis including the lesion characteristics (*p* = 0.865). After adjusting for the FFR value, a multivariable analysis showed that gender (*p* < 0.001), diabetes mellitus (*p* = 0.005), aortic stenosis (*p* = 0.016), hs-CRP level (*p* < 0.001), and eGFR (*p* = 0.003) were all independent predictors of iFR values (Table 3). The fit (*R*^*2*^) of the model was 0.596.

**Table 3:** Determinants of iFR in multivariable regression analysis.

Determinant	Standardized β coefficient	*p*
**FFR**	0.699	< 0.001
**Log hs-CRP**	−0.134	< 0.001
**Female**	−0.126	< 0.001
**eGFR**	0.105	0.003
**Diabetes mellitus**	−0.094	0.005
**Aortic stenosis**	−0.081	0.016

eGFR, estimated glomerular filtration rate; FFR, fractional flow reserve; hs-CRP, high-sensitive C-reactive protein; iFR, instantaneous wave-free ratio.

### Clinical outcomes stratified by FFR and iFR

**Fig 1:**
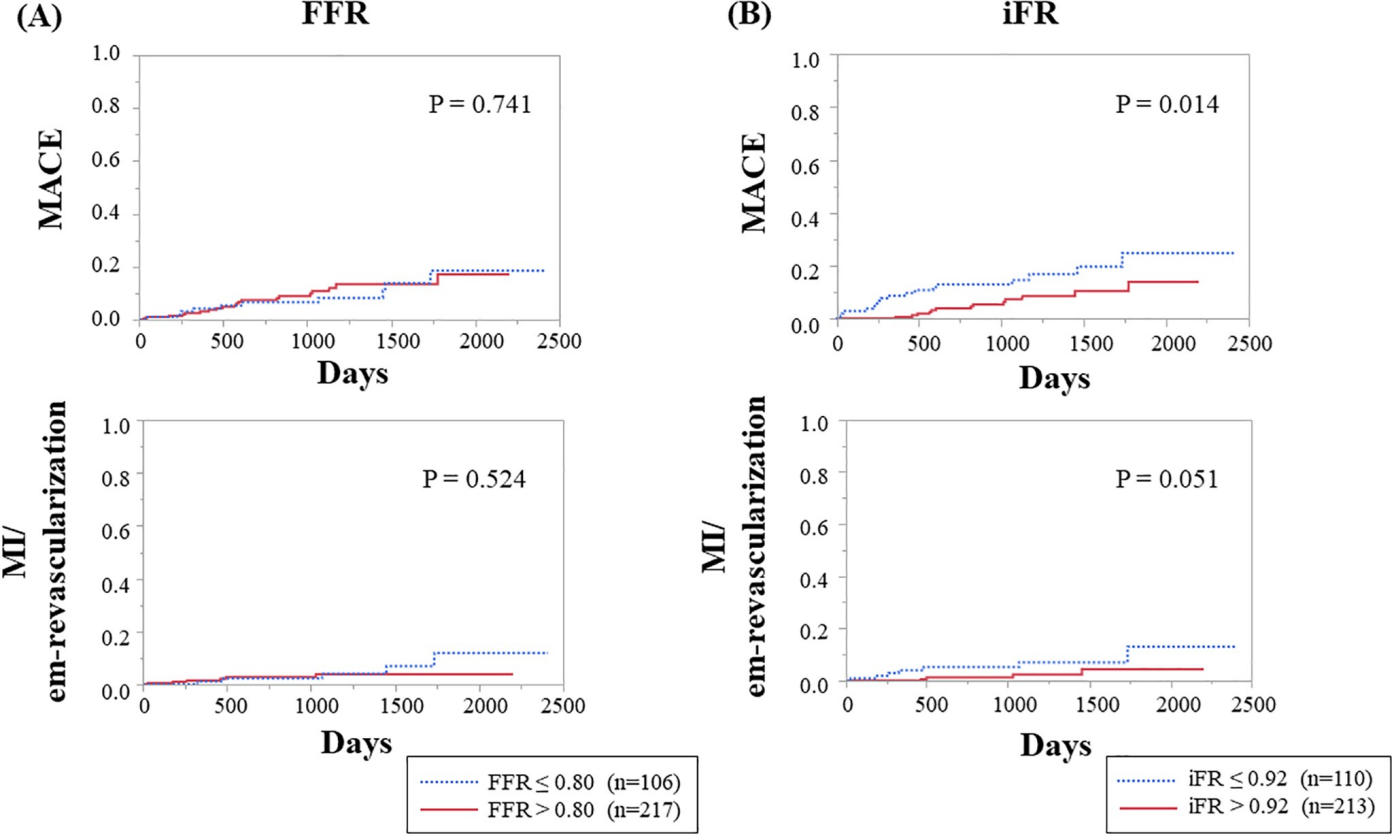
Kaplan-Meier analysis for rates of MACE and MI/need for emergent revascularization according to baseline FFR and iFR values. (A) FFR of ≤ 0.80 versus > 0.80. (B) iFR of ≤ 0.92 versus > 0.92. FFR, fractional flow reserve; iFR, instantaneous wave-free ratio; MACE, major adverse cardiovascular event; MI, myocardial infarction.

In the Kaplan-Meier analysis during a median follow-up of 978 days, the patients with baseline FFR of 0.80 or less did not show worse outcomes with regard to MACE and MI/need for emergent revascularization regardless of subsequent revascularization (Fig 1A). In contrast, those with iFR of 0.92 or less had significantly worse outcomes with regard to MACE (HR, 2.40; 95% CI, 1.16–4.93; *p* = 0.018) and a trend toward more MI and need for emergent revascularization (HR, 3.18; 95% CI, 0.93–10.87; *p* = 0.065) (Fig 1B). In the analysis of the relationship between FFR and iFR, the patients with mismatch between them showed frequent MACE (HR, 2.38; 95% CI, 1.09–5.19; *p* = 0.030) and those with FFR higher than 0.80 and iFR of 0.92 or less had more frequent adverse outcomes than did the other three groups (S1 Table). In Kaplan-Meier analysis, the baseline iFR with cut-off of 0.89 still predicted the MACE and MI/need for emergent revascularization (HR, 2.32; 95% CI, 1.10–4.88; *p* = 0.026 and HR, 4.79; 95% CI, 1.46–15.72; *p* = 0.010, respectively) (S4A Fig).

**Fig 2:**
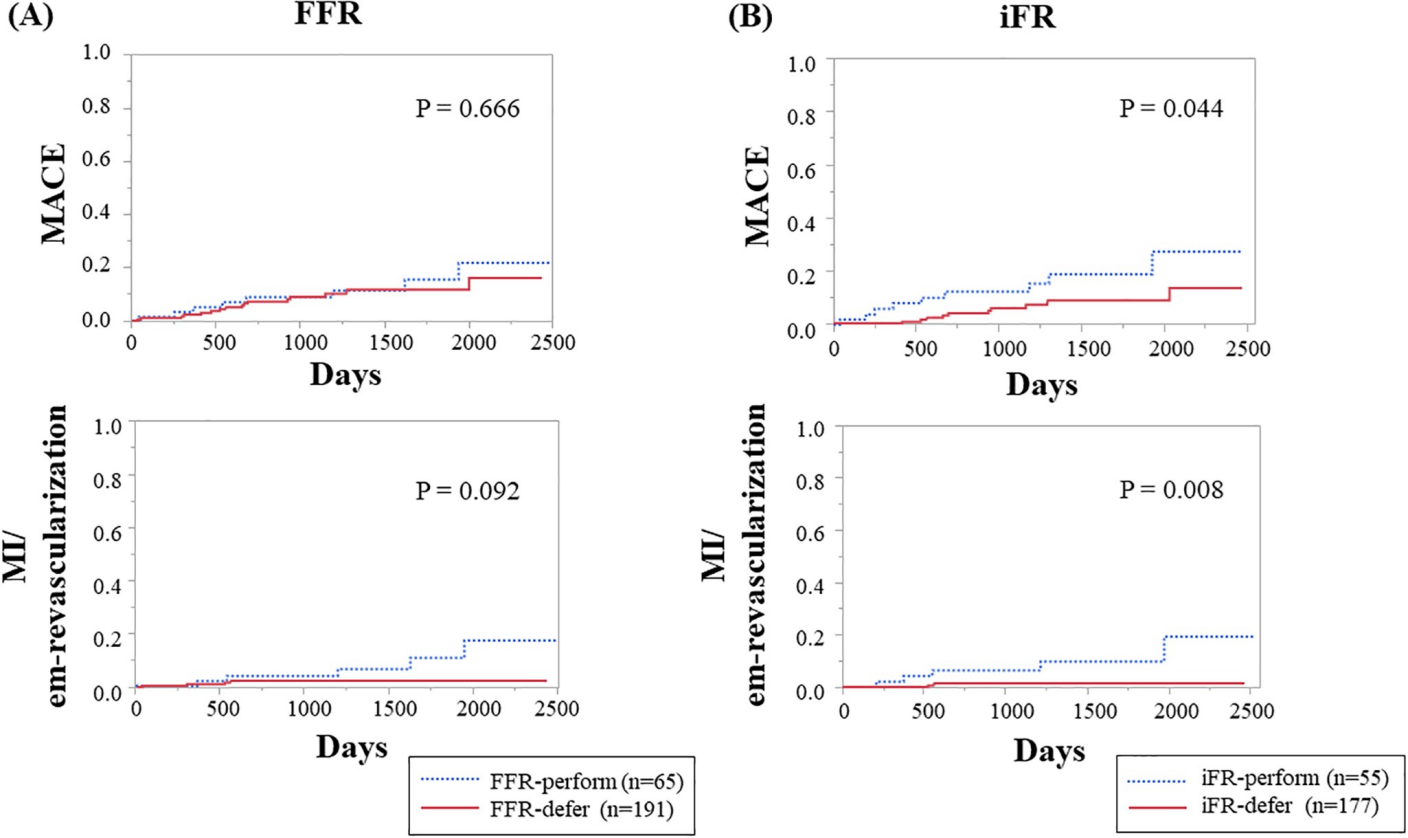
Kaplan-Meier analysis for rates of MACE and MI/need for emergent revascularization according to the treatment strategy. “FFR-defer” and “iFR-defer” groups consisted of patients who did not undergo subsequent revascularization; “FFR-perform” and “iFR-perform” groups consisted of patients who did, on the basis of FFR or iFR values. (A) FFR-perform group versus FFR-defer group. (B) iFR-perform group versus iFR-defer group. FFR, fractional flow reserve; iFR, instantaneous wave-free ratio; MACE, major adverse cardiovascular event; MI, myocardial infarction.

Of the 323 patients, 91 underwent target vessel revascularization (PCI or CABG). After excluding 67 patients who were not treated on the basis of the FFR value, we divided 256 patients into the FFR-defer group (*n* = 191) and the FFR-perform group (*n* = 65; S5A Fig). Similarly, after excluding 91 patients, we divided 232 into the iFR-defer group (*n* = 177) and the iFR-perform group (*n* = 55; S5B Fig). In the Kaplan-Meier analysis, the rates of MACE in the FFR-defer and FFR-perform groups were similar, and the FFR-defer group had lower rates of MI and need for emergent revascularization than did the FFR-perform group (Fig 2A). In contrast, significant differences in the rates of MACE and MI/need for emergent revascularization were observed between the iFR-defer and iFR-perform groups (Fig 2B). In multivariable Cox proportional analysis, after adjusting for age and sex, the FFR-defer group did not have fewer MACE and less MI / need of emergent revascularization than did the FFR-perform group. Moreover, the iFR-defer group had fewer MACE and less MI/need for emergent revascularization than did the iFR-perform group (S2 Table). When the cutoff value of iFR was set to 0.89, the iFR-defer group and the iFR-perform group consisted of 203 and 33 patients, respectively, and in the Kaplan-Meier analysis, significant differences in the rates of MACE and MI/need for emergent revascularization were still observed between the iFR-defer and iFR-perform groups (S4B Fig).

## Discussion

### FFR–iFR mismatch in intermediate coronary artery stenosis

On the basis of the FFR threshold of 0.80, the optimal cutoff of iFR in our cohort was 0.92. At this cutoff, the sensitivity was 74% and the specificity was 86%. Lower cutoff values were reported previously; for example, Götberg et al., Petraco et al., and Ding et al. demonstrated optimal cutoff values of 0.89, 0.90 and 0.91, respectively [6, 16, 17]. The higher optimal cutoff value in our study cohort may be explained by the presence of lower grade ischemia than in the previous studies. The theoretical adoption of a hybrid iFR-FFR strategy provided significantly better results than did a dichotomous cutoff value of 0.90 [18]. In this strategy, iFR values between 0.86 and 0.93 are considered the indeterminate range and values greater than 0.93 indicated nonsignificant ischemia. The latter value is similar to our cutoff value, and it may have resulted in better outcomes with the iFR-defer strategy. However, when the cutoff value of iFR was set to 0.89 in the present cohort, although the lesions with positive ischemia by iFR were decreased and the mismatch rate was slightly increased, the baseline iFR with cut-off of 0.89 still predicted the MACE and the iFR-guided strategy based on this value was still superior. It may suggest that iFR has associations with clinical outcomes regardless of the cutoff value.

FFR and iFR have been demonstrated to show no significant differences in the prediction of myocardial ischemia [4], and a meta-analysis demonstrated excellent agreement of iFR with FFR [5]. However, FFR-iFR mismatch has been recognized, and the reasons for the discrepancies between FFR and iFR remain unknown [8]. In our study, iFR did not match FFR (≤ 0.80) in 18.1% of patients at the optimal cutoff, 0.92. In exploring clinical factors that may explain the discordance between FFR and iFR, we found that gender, diabetes mellitus, hs-CRP level, aortic stenosis, and renal function were independently associated with iFR but not with FFR.

Several researchers have explored the factors associated with the discordance [8, 9]; Lee et al. showed that female gender, diabetes mellitus, smaller reference vessel diameter, and higher percentage DS were associated with low iFR-high FFR discordance. Scarsini et al. reported that the best cutoff for iFR to predict FFR of 0.8 or less was lower in patients with aortic stenosis than in those with CAD [19], which was concordant with our result (optimal iFR cutoffs, 0.90 and 0.93 in patients with and without aortic stenosis, respectively).

### iFR and the physiological significance

Petraco et al. explored the relationship of coronary flow reserve (CFR) with iFR and FFR [20]. iFR showed stronger correlation and better agreement with CFR than with FFR, particularly in the intermediate zone. Cook et al. reported that FFR did not match iFR in 14% of patients and that the disagreement was explained by differences in hyperemic coronary flow velocity and CFR [9]. As mentioned, we found that the clinical factors such as gender, diabetes mellitus, hs-CRP level, aortic stenosis, and renal function, were associated with iFR, independently of FFR. In view of the association of these conditions with impaired microvascular function [21–24], iFR may be more influenced by CFR and may not match FFR in such complex pathophysiological conditions. Another study demonstrated that discrepancies between FFR and iFR might be rationalized by differences in E/e´ on tissue Doppler echocardiography [25]. The diastolic dysfunction shown by increased E/e´ may also be associated with impaired microvascular function or impaired CFR.

### iFR and the clinical outcomes

Two large randomized controlled trials recently demonstrated that revascularization guided by iFR was comparable with that guided by FFR with regard to rates of MACE 1 year later [6, 7]. Escaned et al. performed a post hoc analysis of the pooled data and showed that clinical outcomes for both iFR- and FFR-deferred populations were similar despite a higher rate of deferral with iFR (50% vs. 45% with FFR; *p* < 0.01) [26]. In contrast, Lee et al. investigated 2-year clinical outcomes of FFR- and iFR-guided deferral [27]. Both methods showed a significant association with 2-year rates of MACE. However, assessing the long-term clinical outcomes of the iFR-FFR discordant lesions according to treatment strategy has not been performed or warranted. This is of particular concern in patients with mild to intermediate lesions, such as our cohort. In this study, MACE was associated with lower iFR but not with lower FFR. The patients in whom FFR was greater than 0.80 and iFR was 0.92 or less showed the worst prognosis, which suggests that iFR provided significant prognostic information apart from FFR.

In addition, deferral of revascularization on the basis of iFR greater than 0.92 was associated with fewer MACEs than revascularization that was based on iFR of 0.92 or less, in contrast to the findings with FFR. These findings may suggest that the treatment strategy (deferral or revascularization) should be based on iFR rather than FFR in patients with mild to intermediate coronary stenosis [28]. A prospective validation study is necessary for confirming our findings and their clinical relevance.

### Study limitations

This study had several limitations. First, this was a single center study with a retrospective analysis. Especially, some bias may affect the results of clinical outcomes since some target-vessel revascularizations were performed not on the basis of the FFR value, but on the operators’ decision in some patients. Second, it was up to the operator to decide whether to perform the FFR measurement. The selection bias might be an important limitation. However, in this study, only consecutive lesions of 30% to 80% DS demonstrated on angiography were analyzed, and for such lesions, FFR is routinely measured in our cath lab. Finally, whereas FFR value was calculated on site, iFR value was calculated on a post hoc basis in this study. Although the calculation and analysis were performed in a blind manner, this might have affected the difference in the clinical outcomes by the treatment strategies. Also, iFR was calculated by our own algorithm and not validated with commercially available ones in the present study. It may have had some impact on the threshold of iFR or the match rate with FFR.

## Conclusions

In 323 consecutive patients with CAD and mild to intermediate coronary artery stenosis, iFR-FFR mismatch was observed to a certain extent, and the predictors of iFR, which included clinical factors such as gender, diabetes mellitus, hs-CRP level, renal function, and aortic stenosis, were different from those of FFR. The baseline iFR showed prognostic capacity regardless of subsequent revascularization, and the iFR-guided strategy for predicting cardiac events was clinically superior to the FFR-guided strategy in this setting. These findings may be explained, at least in part, by contribution of the clinical factors on iFR value. The iFR may provide more information about coronary flow status with regard to ischemia and prognostic information than does FFR in patients with mild to intermediate CAD.

## Supporting information

S1 Fig Patient enrollment in this study. CAG, coronary angiography; FFR, fractional flow reserve; iFR, instantaneous wave-free ratio.
(TIF)

S2 Fig Receiver operating characteristics analysis for iFR as an indicator of ischemia on the basis of FFR value no more than 0.80. AUC, area under the curve; FFR, fractional flow reserve; iFR, instantaneous wave-free ratio.
(TIF)

S3 Fig Relationship between FFR and iFR. Each red dot denotes a patient with a major cardiac event; each black dot denotes a patient with no major cardiac event. FFR, fractional flow reserve; iFR, instantaneous wave-free ratio; N, number.
(TIF)

S4 Fig Kaplan-Meier analysis for rates of MACE and MI/need for emergent revascularization according to baseline iFR values and the treatment strategy on the basis of the cutoff vale of iFR: 0.89. (A) iFR of ≤ 0.89 versus > 0.89. (B) iFR-perform group versus iFR-defer group. FFR, fractional flow reserve; iFR, instantaneous wave-free ratio; MACE, major adverse cardiovascular event; MI, myocardial infarction.
(TIF)

S5 Fig Patient enrollment in the prognosis analysis. “FFR-defer” and “iFR-defer” groups consisted of patients who did not undergo subsequent revascularization; “FFR-perform” and “iFR-perform” groups consisted of patients who did, on the basis of FFR or iFR values. FFR, fractional flow reserve; iFR, instantaneous wave-free ratio; MACE, major adverse cardiovascular event.
(TIF)

S1 Table Clinical outcomes according to FFR and iFR values. FFR, fractional flow reserve; iFR, instantaneous wave-free ratio; MACE, major adverse cardiovascular event; MI, myocardial infarction.
(DOCX)

S2 Table Cox multivariable models to identify hazard ratio of clinical outcomes. *Adjusted for age and sex. “FFR-defer” and “iFR-defer” groups consisted of patients who did not undergo subsequent revascularization; “FFR-perform” and “iFR-perform” groups consisted of patients who did, on the basis of FFR or iFR values. FFR, fractional flow reserve; iFR, instantaneous wave-free ratio; MACE, major adverse cardiovascular event; MI, myocardial infarction.
(DOCX)

S1 Dataset (XLSX)

S2 Dataset (XLSX)
