# Supplementary figures and images for: Determinants and prognostic implications of instantaneous wave-free ratio in patients with mild to intermediate coronary stenosis: Comparison with those of fractional flow reserve
Source: PLoS One. 2020 Aug 6;15(8):e0237275. doi: 10.1371/journal.pone.0237275 (PMC7410195; doi:10.1371/journal.pone.0237275)

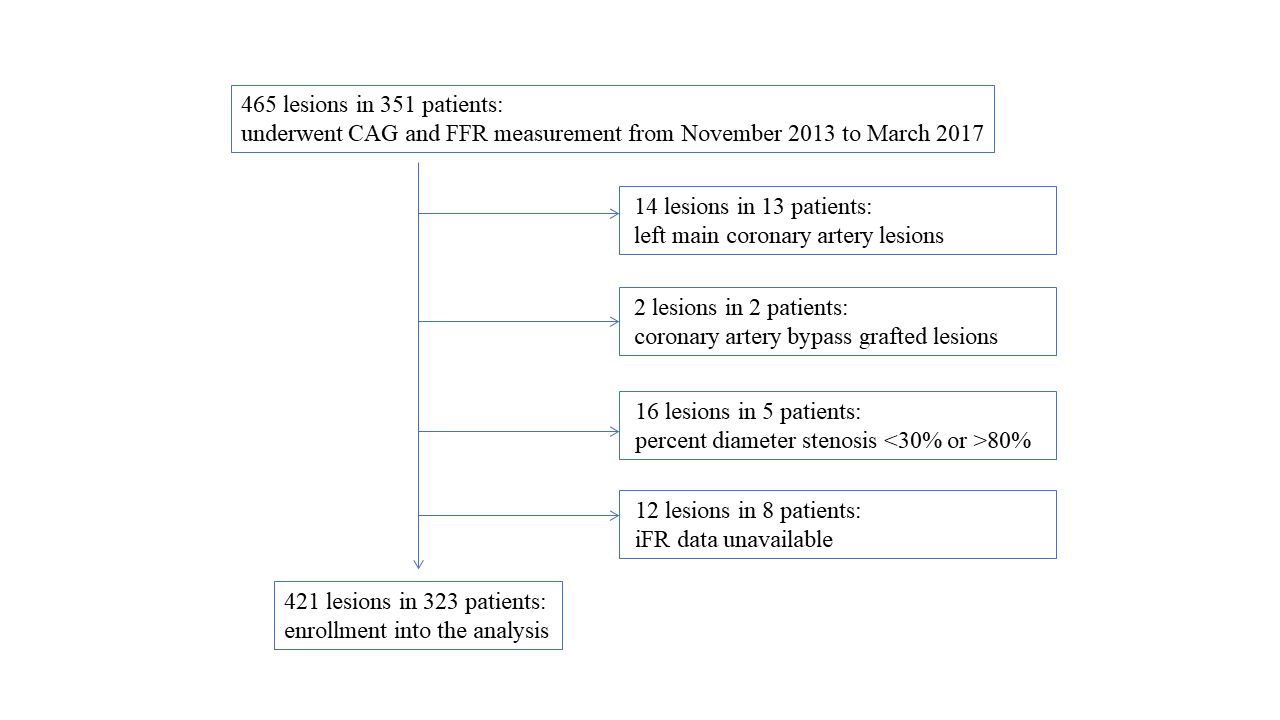

Supplement: S1 Fig — CAG, coronary angiography; FFR, fractional flow reserve; iFR, instantaneous wave-free ratio. (TIF) [file pone.0237275.s001.TIF]

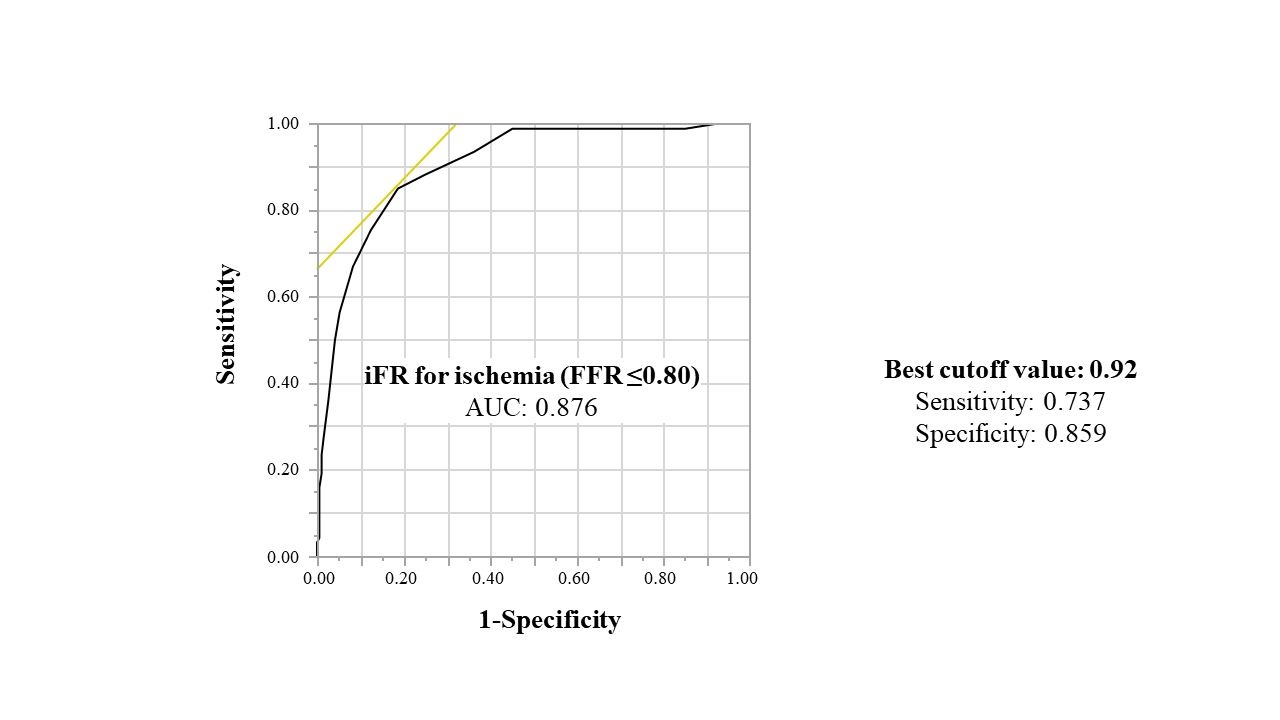

Supplement: S2 Fig — AUC, area under the curve; FFR, fractional flow reserve; iFR, instantaneous wave-free ratio. (TIF) [file pone.0237275.s002.TIF]

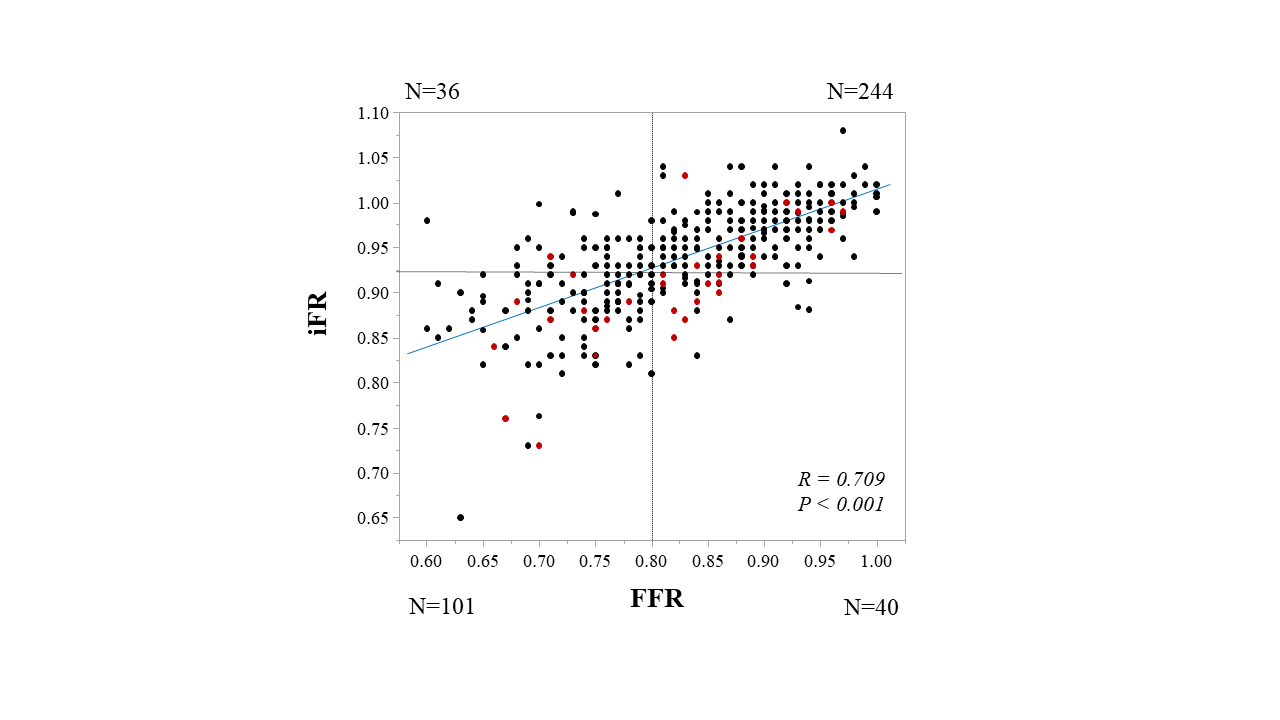

Supplement: S3 Fig — Each red dot denotes a patient with a major cardiac event; each black dot denotes a patient with no major cardiac event. FFR, fractional flow reserve; iFR, instantaneous wave-free ratio; N, number. (TIF) [file pone.0237275.s003.TIF]

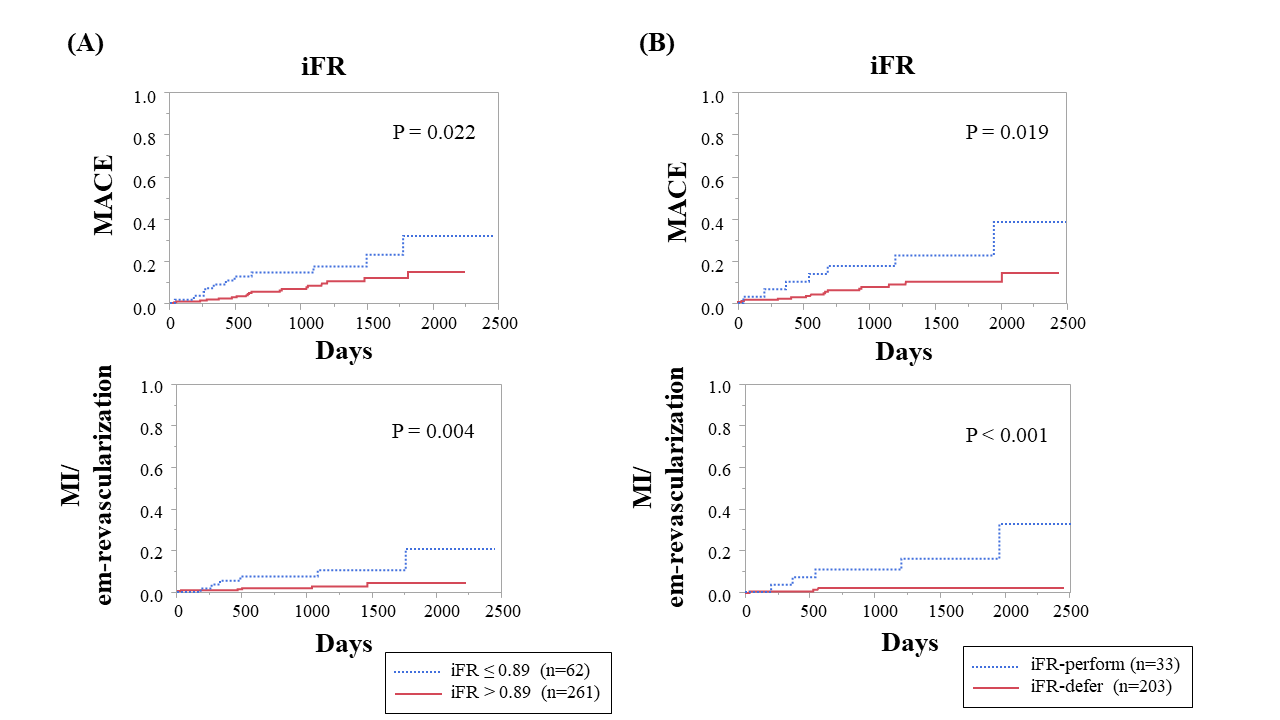

Supplement: S4 Fig — (A) iFR of ≤ 0.89 versus > 0.89. (B) iFR-perform group versus iFR-defer group. FFR, fractional flow reserve; iFR, instantaneous wave-free ratio; MACE, major adverse cardiovascular event; MI, myocardial infarction. (TIF) [file pone.0237275.s004.TIF]

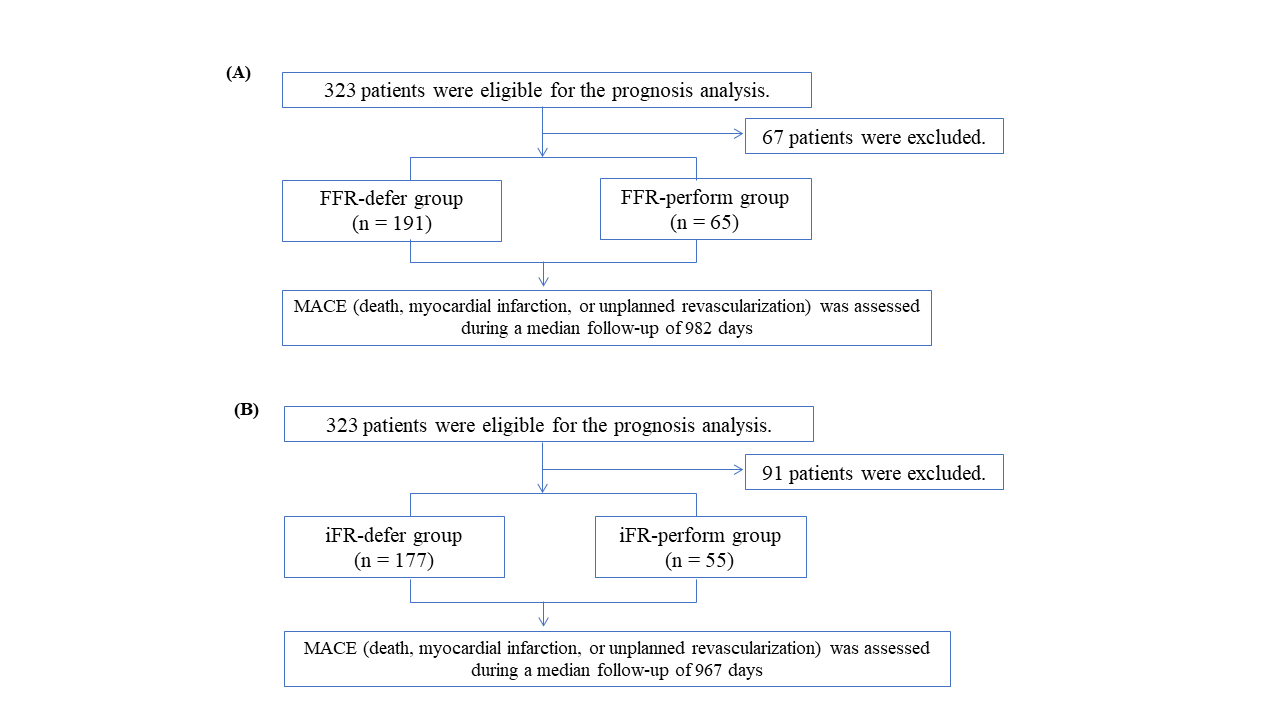

Supplement: S5 Fig — “FFR-defer” and “iFR-defer” groups consisted of patients who did not undergo subsequent revascularization; “FFR-perform” and “iFR-perform” groups consisted of patients who did, on the basis of FFR or iFR values. FFR, fractional flow reserve; iFR, instantaneous wave-free ratio; MACE, major adverse cardiovascular event. (TIF) [file pone.0237275.s005.TIF]
