# Supplementary material for: Determinants and prognostic implications of instantaneous wave-free ratio in patients with mild to intermediate coronary stenosis: Comparison with those of fractional flow reserve
Source: PLoS One. 2020 Aug 6;15(8):e0237275. doi: 10.1371/journal.pone.0237275 (PMC7410195; doi:10.1371/journal.pone.0237275)
Supplement: S1 Table — FFR, fractional flow reserve; iFR, instantaneous wave-free ratio; MACE, major adverse cardiovascular event; MI, myocardial infarction. (DOCX) [file pone.0237275.s006.docx]

| **Event** | **FFR≤0.80**  **iFR≤0.92** | **FFR>0.80**  **iFR>0.92** | **FFR≤0.80**  **iFR>0.92** | **FFR>0.80**  **iFR≤0.92** |
| --- | --- | --- | --- | --- |
| **Number of lesions** | 82 | 189 | 24 | 28 |
| **MACE (%)** | 11.0 | 6.4 | 4.2 | 28.6 |
| **MI/revascularization (%)** | 4.9 | 1.6 | 4.2 | 10.7 |
