# Supplementary material for: Determinants and prognostic implications of instantaneous wave-free ratio in patients with mild to intermediate coronary stenosis: Comparison with those of fractional flow reserve
Source: PLoS One. 2020 Aug 6;15(8):e0237275. doi: 10.1371/journal.pone.0237275 (PMC7410195; doi:10.1371/journal.pone.0237275)
Supplement: S2 Table — *Adjusted for age and sex. “FFR-defer” and “iFR-defer” groups consisted of patients who did not undergo subsequent revascularization; “FFR-perform” and “iFR-perform” groups consisted of patients who did, on the basis of FFR or iFR values. FFR, fractional flow reserve; iFR, instantaneous wave-free ratio; MACE, major adverse cardiovascular event; MI, myocardial infarction. (DOCX) [file pone.0237275.s007.docx]

| **Outcomes** | **Adjusted* hazard ratio** | **95% confidence interval** | ***p*** |
| --- | --- | --- | --- |
| FFR-defer vs. FFR-perform |  |  |  |
| MACE | 0.740 | 0.306–1.793 | 0.505 |
| MI/revascularization | 0.316 | 0.078–1.276 | 0.106 |
| iFR-defer vs. iFR-perform |  |  |  |
| MACE | 0.407 | 0.163–1.015 | 0.054 |
| MI/revascularization | 0.137 | 0.025–0.743 | 0.021 |
